# Integrated multi-omics analyses reveal the pleiotropic nature of the control of gene expression by Puf3p

**DOI:** 10.1038/srep15518

**Published:** 2015-10-23

**Authors:** Christopher J. Kershaw, Joseph L. Costello, David Talavera, William Rowe, Lydia M. Castelli, Paul F. G. Sims, Christopher M. Grant, Mark P. Ashe, Simon J. Hubbard, Graham D. Pavitt

**Affiliations:** 1 Faculty of Life Sciences, The University of Manchester, Michael Smith Building, Oxford Road, Manchester, M13 9PT, United Kingdom

## Abstract

The PUF family of RNA-binding proteins regulate gene expression post-transcriptionally. *Saccharomyces cerevisiae* Puf3p is characterised as binding nuclear-encoded mRNAs specifying mitochondrial proteins. Extensive studies of its regulation of *COX17* demonstrate its role in mRNA decay. Using integrated genome-wide approaches we define an expanded set of Puf3p target mRNAs and quantitatively assessed the global impact of loss of *PUF3* on gene expression using mRNA and polysome profiling and quantitative proteomics. In agreement with prior studies, our sequencing of affinity-purified Puf3-TAP associated mRNAs (RIP-seq) identified mRNAs encoding mitochondrially-targeted proteins. Additionally, we also found 720  new mRNA targets that predominantly encode proteins that enter the nucleus. Comparing transcript levels in wild-type and *puf3*∆ cells revealed that only a small fraction of mRNA levels alter, suggesting Puf3p determines mRNA stability for only a limited subset of its target mRNAs. Finally, proteomic and translatomic studies suggest that loss of Puf3p has widespread, but modest, impact on mRNA translation. Taken together our integrated multi-omics data point to multiple classes of Puf3p targets, which display coherent post-transcriptional regulatory properties and suggest Puf3p plays a broad, but nuanced, role in the fine-tuning of gene expression.

Post-transcriptional regulation of mRNA is central to diverse cellular processes and plays an important role in the overall control of gene expression. Indeed, recent estimates of the relative contributions of different steps in gene expression highlight a significant role for mechanisms affecting translation^1^. Post-transcriptional regulation can be achieved by several mechanisms including the recognition of mRNAs by multiple general and specific RNA binding proteins (RBPs) that modulate their fate^2^. RBPs can activate or repress mRNA translation, target mRNAs for degradation or storage, or direct mRNAs to a specific location within a cell^2^,^3^. Puf3p, a member of the PUF domain family, is one of these RNA-binding proteins found across eukarya^4^. PUF proteins can regulate mRNA fate by affecting mRNA-stability and/or the translation of targeted mRNAs^5^,^6^. *S. cerevisiae* has six PUF domain containing proteins, Puf1-6p, that each bind specific sequences, often within 3′ untranslated regions (3′ UTR) via their PUF repeat domains^7^,^8^,^9^.

Puf3p is found distributed throughout the cytoplasm^8^,^10^,^11^ and its mRNA targets have been previously characterised by coupling affinity capture and microarray methodologies (‘RIP-chip′ approach)^8^. RNAs bound to Puf3-TAP were captured via immunoprecipitation on IgG beads (RIP) and subsequently identified by microarrays (chip); 225 mRNAs predominantly encoding mitochondrial targeted proteins were bound significantly, implicating Puf3p in regulating the expression of multiple mitochondrial proteins. The PUF domain comprises eight repeated PUF motifs that combine to recognise a consensus motif: CNUGUAHAUA, where H is either A, C or U^8^,^9^ as shown in crystal structures of the Puf3p RNA-binding domain in concert with *COX17* 3′ UTR sequences^12^. Each PUF repeat forms three alpha helices that binds one nucleotide. Two residues within helix 2 make direct contacts with a single RNA base, while a third amino acid residue is stacked above it^12^,^13^.

Multiple additional experiments show that Puf3p can regulate mitochondrial functions. It has been shown to promote degradation of certain mitochondrial mRNAs^7^, and Puf3p abundance is reduced during diauxic shift and growth on non-fermentable carbon sources when mitochondria are up-regulated^14^. In addition, *PUF3* deletion causes mitochondrial morphological and motility abnormalities^15^, increased cellular oxygen consumption^16^ and is involved in oxidative stress tolerance^17^. Fluorescence microscopy of several mRNAs indicates a role for Puf3p in localising mRNAs. For example, *OXA1*, *IMG1* and *RSM25* have some dependence upon Puf3p for localisation to mitochondria^18^.

The most studied Puf3p target is *COX17* mRNA, encoding a copper metallochaperone that shuttles cytoplasmic copper to mitochondria^19^. *COX17* mRNA contains two Puf3p binding sites in its 3′ UTR and upon binding of Puf3p is targeted to the mitochondria or marked for degradation. Puf3p promotes deadenylation and subsequent decay of *COX17*^7^,^20^ and can interact in an mRNA dependent manner with the members of the mRNA degradation machinery, including members of the Lsm ring, the Ccr4p and Pan2p pathways and the decapping complex^21^,^22^. Hence *COX17* has been a useful model for studies of mRNA decay mechanisms, but it is unclear how typical *COX17* is of Puf3p target mRNAs.

Although Puf3p can act to localise specific mRNAs to mitochondria and to enhance mRNA degradation, recent studies suggest more complex roles for Puf3p. For example, Puf3p was shown to interact with translating ribosomes^17^ and in an RNA-dependent manner with multiple members of the ‘closed loop’ complex^21^. Also a study that used a cross-linking approach to capture Puf3p mRNA targets and then next-generation sequencing ‘PAR-clip’ (photoactivatable-ribonucleoside-enhanced UV cross-linking and immunoprecipitation) identified a much larger set of interacting mRNAs than originally identified by RIP-chip^23^. Interestingly most of the new mRNAs found were not mitochondrial, raising questions as to how comprehensive/selective each study was in identifying Puf3p target mRNAs.

In order to address these questions, we set out to re-evaluate Puf3p mRNA targets and study Puf3p’s wider role in regulating gene expression. For the first time, we combine multiple post-genomics techniques to investigate the global impact of deleting *PUF3* on multiple levels of gene expression, from transcript to proteome. Our genome-wide analyses define an expanded set of Puf3p targets and support a broader perspective on Puf3p activities, and address whether *COX17* is a fully representative target. We conclude that Puf3p can interact with many more mRNAs than previously appreciated, greatly expanding the population of potential Puf3p target mRNAs. However, we find that the steady-state level of only a small fraction of these mRNAs is altered in *puf3∆* cells. In contrast the engagement of many Puf3p-target mRNAs with translating ribosomes is altered. We conclude that although *COX17* is a principal target that is greatly affected by perturbations in *PUF3*, few other Puf3p targets are as dramatically affected by loss of Puf3p, and that it likely delivers its functions via multiple mechanisms.

## Results

### RIP-seq identifies over 1000 Puf3p target mRNAs

Two prior studies have provided different perspectives on the yeast mRNAs associated with Puf3-TAP, a genomically integrated tag allowing affinity purification of the bait protein. Firstly a seminal RIP-chip study used microarray detection to identify 225  mRNAs encoding mostly mitochondrial proteins^8^. In contrast a PAR-clip approach using high-throughput sequencing identified 988  mRNAs, with an overlap of 131 mRNAs identified in both studies and the majority of the novel Puf3p targets identified not encoding mitochondrial proteins^23^. As both studies used the same strains and growth conditions the reasons for the differences were not clear.

**Figure 1:**
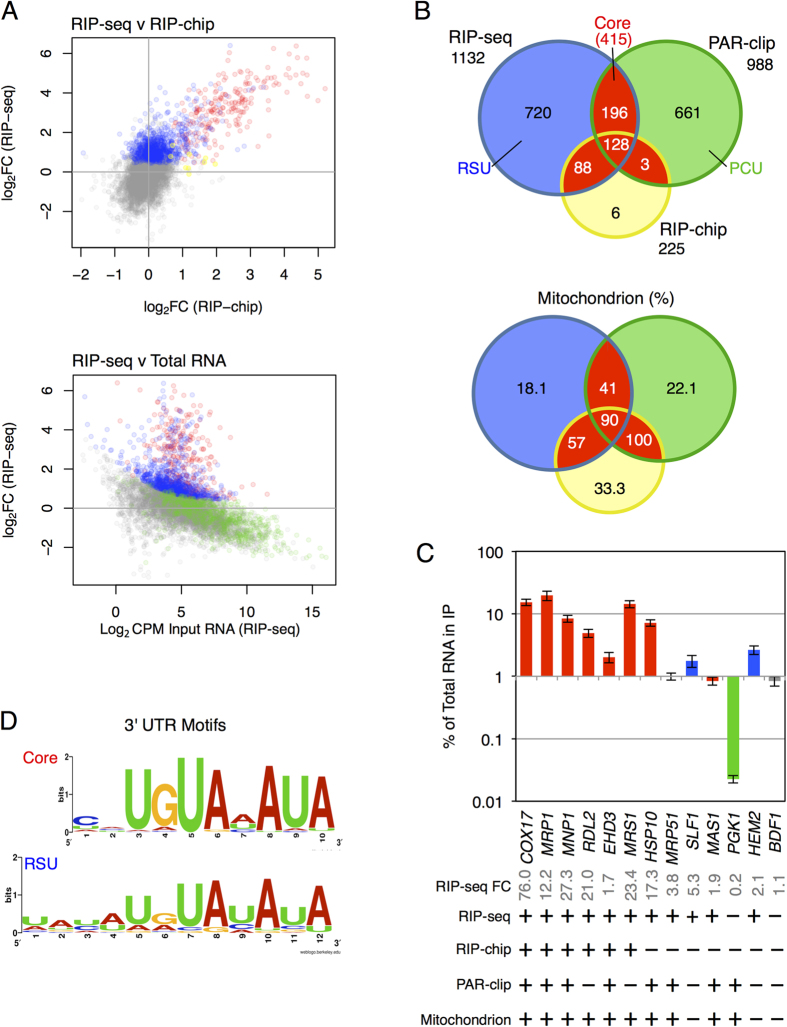
Puf3p RIP Seq identifies novel Puf3p mRNA targets. (**A**) Comparison of our RIP-seq data (blue) with the RIP-chip^9^ (yellow) and PAR-clip^23^ data (green) identify a common Core set of Puf3p target mRNAs identified in multiple studies (Red), with non-targets colored grey. Top, Scatter-plot comparing the log2 fold enrichments (IP/total) of RIP-seq and previously published RIP-chip data^9^. Bottom, plot of log2 fold enrichments (IP/total) versus transcript abundance. (**B**) Venn-style diagrams showing overlaps between our experiment (RIP-seq) and two prior studies. Top chart shows the numbers of RNAs, the lower chart shows the percentage of mitochondrial targets in each sector. Sector colouring follows that used in panel A. (**C**) 13 mRNAs were quantified using qPCR to confirm our RIP-seq analysis. Data shown are a mean of biological triplicates. Error is Standard error of the mean. Using a digital +/− descriptor where characteristics of each ORF tested is described, ORFs significantly enriched in the Puf3 RIP-seq, PAR-clip and RIP-chip are shown. Mitochondrion GO category presence is also indicated. (**D**) Logo plots of the motifs found in each 3′ UTR set using MEME. Further details are in Supplementaary Dataset S1.

In an attempt to reconcile these differences we performed a RIP-seq experiment using glucose synthetic complete medium. To avoid glucose starvation upon sample harvest, cells were washed in medium rather than buffer and rapidly frozen in liquid nitrogen prior to cell lysis and affinity purification. Subsequent data processing of triplicate experiments showed clear clustering of the RIP-seq and total RNA replicates into distinct clusters (Supplementary Figure S1). A total of 1132  mRNAs were significantly enriched (FDR <0.01) with Puf3-TAP over total RNA (Supplementary Dataset S1), more than found previously using microarray detection (RIP-chip)^8^,^9^. Directly comparing these two datasets revealed that 216 of the 225 original targets are enriched in our data set, with high similarity in the mean fold-enrichments pointing to a remarkable convergence in the data. Both studies identify common highly-enriched transcripts (red circles in Fig. 1A, top), while the greater resolving power and dynamic range of the RNA sequencing enabled us to identify additional target mRNAs (blue circles).

Similarly there is large overlap between our data and the PAR-clip study^23^. Comparing all three datasets together reveals there are an additional 196 mRNAs shared between the PAR-clip and our RIP-seq data that were not identified by the earlier RIP-chip analysis (Fig. 1B). Those mRNAs uniquely identified only in the PAR-clip study^23^ (coloured green in Fig. 1A and B, and defined here as PAR-clip Unique or PCU) appear depleted in our RIP-seq data and are enriched for higher abundance transcripts, while our novel targets (termed RIP-seq unique, RSU) include lower abundance transcripts (Fig. 1A, lower panel; Supplementary Figure S2D). Of the 415 transcripts present in two or more datasets, 60% are nuclear-encoded mitochondrial mRNAs (Fig. 1B, lower panel). This suggests that these 415 mRNAs represent a set of ‘Core’ targets, which likely share common functional properties, and so have been defined as such for the remainder of this manuscript. A complete list of these mRNAs is provided (Supplementary Dataset S1).

To address whether the 720 RSU targets represented *bona fide* Puf3p candidates or were enriched in our data for other reasons we performed a series of control experiments which strongly suggest that they are specific interacting mRNAs and are not enriched by virtue of indirect interactions (Supplementary Text S1 and Supplementary Figure S2). As a final independent validation of our RIP-seq results, we performed qRT-PCR analysis on a representative selection of mRNAs (Fig. 1C). Core Puf3p targets (*COX17*, *MRP1*, *MNP1*, *RDL2*, *EHD3* and *MRS1*) were bound by Puf3p whereas other control mRNAs (*PGK1* and *BDF1*) were not. *PGK1* was identified in the PAR-clip study, but is not significantly bound in this experiment. Importantly, novel RSU targets, *HEM2* and *SLF1*, were confirmed (Fig. 1C).

### New mRNA targets are enriched with the Puf3p binding motif

Puf3p-bound mRNAs typically possess a conserved motif, most frequently within their 3′ UTRs, but also noted in a few coding regions and some 5′ UTRs^9^,^23^. RIP-seq experiments do not capture the RNA fragments specifically bound by the RBP. To assess the various subsets of Puf3p-target mRNAs for possible Puf3p binding motifs, we used a computational approach, screening for common motifs within the 5′ and 3′ UTRs using the MEME tool^24^. Using this *ab initio* approach we found that the 415 Core set was highly enriched for a [CU]HUGUA[AU]AUA binding motif in 3′ UTR sequences, virtually identical to previously reported Puf3p motifs^12^, CNUGUAHAUA (Fig. 1D). Further examination of our novel 720 RSU 3′ UTRs identified a Puf3p motif enriched in all sequences tested that is essentially identical to the Core and previously reported motifs, with a minor 5′ [UA]A extension [UA]A[UC][AU]U[GA]UA[UC]AUA. The presence of the Puf3p binding motif provides further support to the assertion that they represent genuine novel Puf3p targets.

### Functional classification of Puf3p targets suggests it has both nuclear and mitochondrial roles.

In agreement with prior studies, Gene Ontology (GO) analyses of the three groups of Puf3p-target mRNAs revealed enrichment for mitochondrial function. In total 60% of the core mRNA targets encode mitochondrially functioning proteins, with further mitochondrial proteins within the PCU and the RSU sets; 22% and 18% respectively (Fig. 1B, lower panel). Our RIP-seq adds 132 genes to the list of mitochondrial targets, while combining all three studies indicates that Puf3p binds almost half (48.4%) of the 1086 GO-annotated mitochondrial proteins.

**Figure 2:**
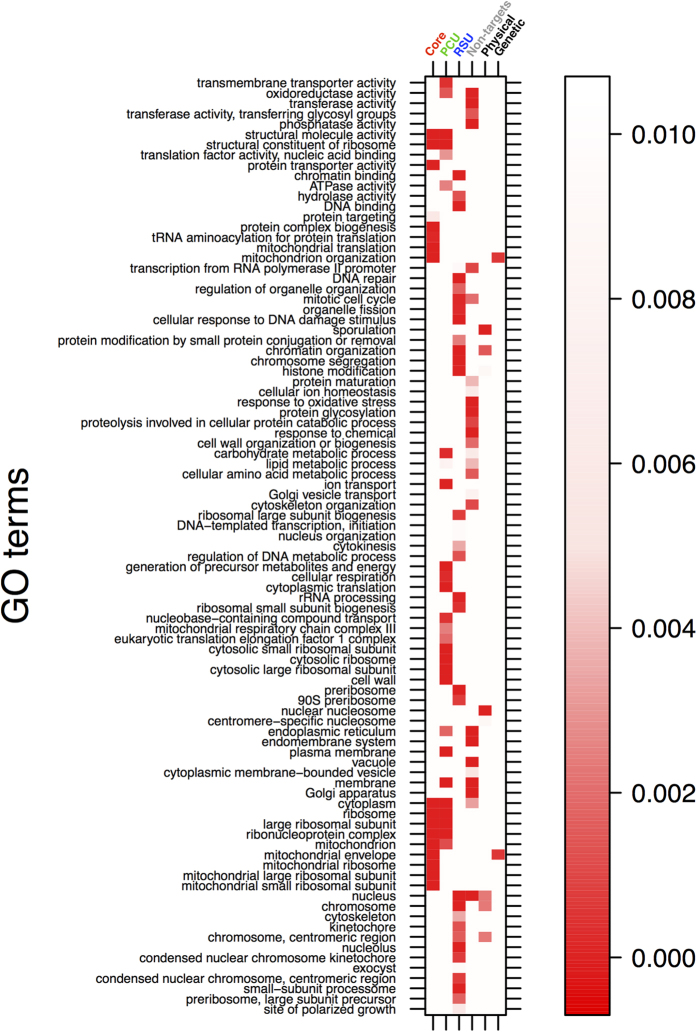
GO term enrichment of Puf3p target mRNAs. GO term enrichment of those mRNAs that are enriched in the Core, RSU, PCU datasets as well as non-targets, genetic and physical interactors of Puf3p as determined by the BioGRID database^25^. Red shading intensity denotes significance (FDR) of enrichment according to the adjacent key.

GO analysis also reveals RSU targets display significant enrichment for ribosome and pre-ribosome components along with multiple nuclear functions (Fig. 2). These enrichments are distinct from non-target mRNAs. We also analysed the GO categories of direct (first-order) Puf3p physical and genetic interactors, as reported in BioGRID^25^ because these interactions can provide important insight in the possible roles of Puf3p. Interestingly, genetic interactors were enriched for mitochondrial function, while physical interactions were enriched for nuclear activity/location (Fig. 2). These data suggest that in addition to its role regulating mRNAs encoding proteins functioning in mitochondria, Puf3p may regulate proteins functioning in the nucleus and nucleolus, possibly as an mRNA-binding component of multi-protein complexes.

### Deletion of *PUF3* affects the mRNA steady state abundance of only a small fraction of Puf3p target mRNAs

To assess whether Puf3p was likely to have a broad influence on the mRNA stability of its targets, we compared polyA tail length^26^ and mRNA stability half-lives from genome-wide studies conducted in wild-type cells^27^ (Supplementary Figure S3). We found that Puf3p-target mRNAs have significantly shorter polyA tails than non-targets, and the RSU set also has significantly shorter mRNA half-lives (Supplementary Figure S3). While not conclusive these observations are consistent with a known role for Puf3p in polyA shortening^7^, one step during mRNA decay.

Prior candidate gene studies have examined the stability of selected Puf3p target mRNAs using a temperature sensitive RNA polymerase II *rpb1-1* allele^20^,^28^. Recently *COX17* and ten additional mRNAs were studied that were found to be 1.5–4 fold more stable in *puf3*∆ versus *PUF3* glucose-grown strains. In contrast the earlier report found two Puf3p target mRNAs whose stability was not affected by *puf3*∆^28^. These data suggest that controlling mRNA stability is a major function of Puf3p in glucose grown cells; however, no genome-wide study has yet examined the impact of *puf3∆* in glucose grown cells across either the transcriptome or proteome. We therefore decided to characterise the effects of *puf3*∆ globally across multiple gene expression stages.

**Figure 3:**
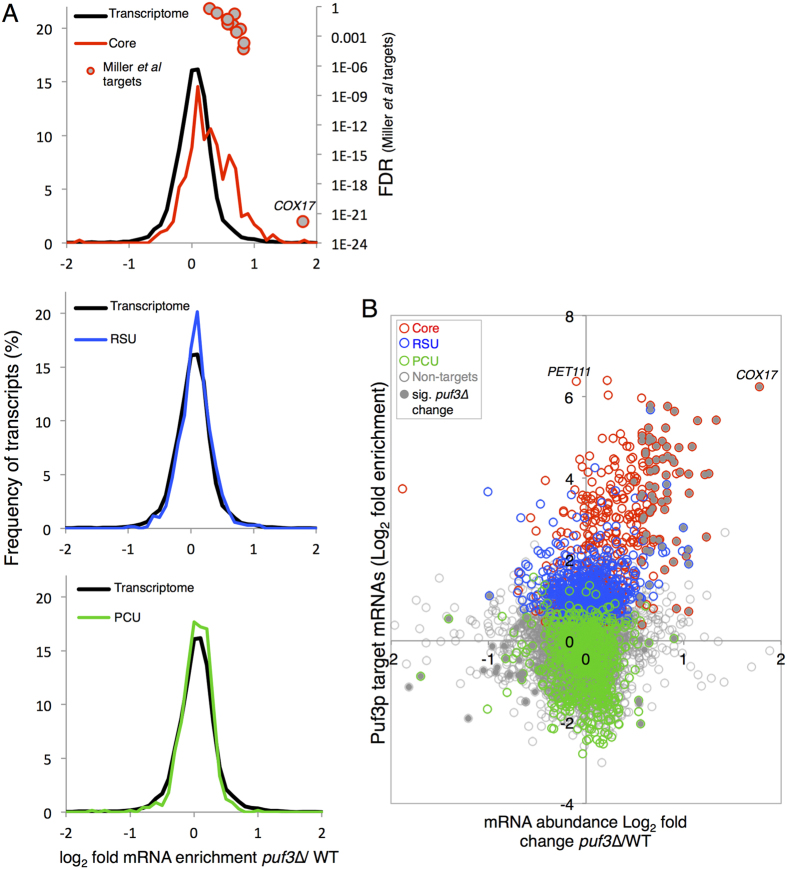
Puf3p affects the abundance of only some of its target mRNAs. (**A**) Relative transcript abundance changes Log_2_ fold enrichment *puf3*Δ/Wild type. Transcriptome changes were split into ‘bins’ (0.25 fold/bin) and expressed as a percentage of transcripts in each bin for the Core targets (red), RSU (blue) and PCU (green) as defined in Fig. 1A. mRNAs whose stability has been previously shown to be affected by a deletion of *PUF3*^20^ are all Core targets and are also plotted (red circle, grey filled). (**B**) A scatterplot comparing the mRNA abundance in the *puf3*Δ mutant strain and Puf3p mRNA targets identified by RIP Seq. Those that change significantly (FDR < 0.05) have grey filled symbols.

Transcript abundance was determined by SOLiD RNA sequencing of isolated total RNA from biological triplicates of both *puf3*Δ and its parental strain. Only 82 mRNAs significantly increase (FDR<0.05) in abundance in *puf3*Δ strains and this includes 8 of the 11 mRNAs previously shown to be stabilized by *puf3*∆ (Fig. 3A, top panel filled circles)^20^. Notably, the well-known Puf3p target *COX17* changed most, with a three-fold relative increase in abundance, similar to the levels when mRNA stability was measured directly. More broadly, however, we were surprised that relatively few mRNA levels changed between the two strains (Supplementary Dataset S1). Of the 82 transcripts that increase in the *puf3*Δ strain, all but three (*AAP1*, *COX6* and *QCR2*) were identified as Puf3p targets in at least one of the three RNA-interaction studies and two of these non-targets (*COX6* and *QCR2*) encode mitochondrial proteins. Thus 80/82 transcripts that increase in *puf3*Δ strains are mitochondrial, expanding and reinforcing the link between Puf3p and control of mitochondrial activity. The two non-mitochondrial targets, *HEM2* and *AAP1*, are annotated as encoding cytoplasmic and nuclear proteins. Our qPCR validated *HEM2* as a Puf3p-target (Fig. 1D).

Although steady-state measurements of RNA abundance depend on both synthesis and decay, as very few mRNA levels are altered, these analyses suggest that the stability of only a fraction of Puf3p-bound mRNAs is impacted by loss of Puf3p (Fig. 3A). The same general observation was made for array-based Puf3p targets in a *puf3*Δ strain grown in glycerol^8^. In contrast, non-Core target mRNAs do not alter in abundance compared to the total transcriptome. By comparing the transcriptome of *puf3*Δ strains to the fold enrichment in our RIP-seq experiment a number of points are clear (Fig. 3B). Firstly, it shows that *COX17*, rather than being a typical Puf3p target mRNA, is one of the most enriched mRNAs in the Puf3p IP and is by far the most increased in transcript abundance after deletion of *PUF3*. Secondly there is no strict correlation between Puf3p binding *per se* and altered mRNA abundance in *puf3*Δ strains. For example, *PET111* is a Core target that is 83-fold enriched in our RIP-seq, similar to *COX17*, but the mRNA abundance is unaltered in a *puf3*Δ strain. Many other mRNAs clearly also fall on, or close to, zero on the x-axis. This global analysis of mRNA levels in *puf3∆* suggests that although some Puf3p target mRNAs increase in abundance after deletion of *PUF3*, Puf3p is not likely a rate-limiting factor for degradation of the majority of its targets.

### The impact of *PUF3* deletion on the proteome

**Figure 4:**
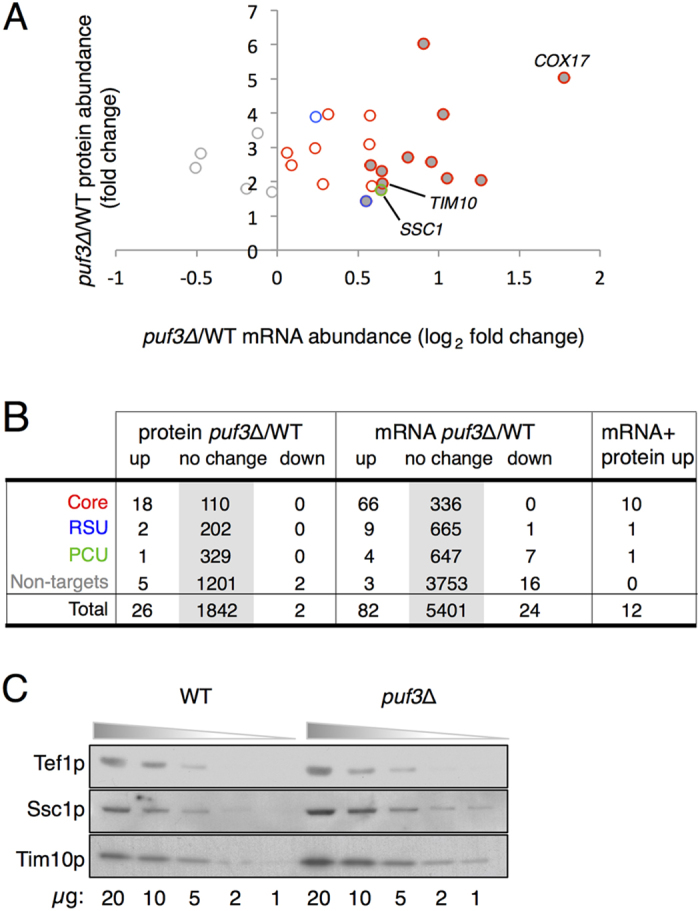
Proteomic analysis of *puf3*Δ and wild-type strains. (**A**) Fold-enrichment for proteins identified as increasing or decreasing in the *puf3*Δ mutant compared with the wild-type strain (Y-axis) plotted with changes in mRNA abundance from RNA-seq (X-axis). Only proteins found to significantly alter in abundance (FDR < 0.05) are shown. Data points are coloured as per Fig. 1. Proteins whose mRNAs are also altered in abundance are filled grey. (**B**) Summary table comparing proteome transcriptome and Puf3p interactions. (**C**) Immunoblotting of Tef1p (a loading control), Tim10p and Ssc1p. The indicated amount of total soluble protein (μg) was loaded from *puf3*Δ and wild-type strains.

As a putative translational regulator, we determined the impact that the loss of Puf3p has on the proteome using label-free quantitative mass spectrometry on whole cell extracts. Five replicates of wild-type and *puf3*∆ strains grown in conditions identical to our RNA-seq experiments were analysed via LC-MS/MS. This identified 2103 yeast proteins of which 1870 yielded quantitative information using a Progenesis workflow (Supplementary Dataset S1). Although coverage is not complete, 662/1870 proteins are within the three Puf3p-target datasets. Surprisingly only 28 proteins were significantly altered in abundance in *puf3*∆ cells (FDR < 0.05); 26 increase and two decrease in *puf3*Δ cells (Fig. 4A,B). Neither of the mRNAs encoding the two decreasing proteins (Cdc10p and Tgl1p) were Puf3p bound. In contrast 21/26 proteins that increase were encoded by mRNAs enriched in at least one Puf3-IP study (Fig. 4A,B). Comparing our transcriptomic *versus* proteomic analyses reveals fewer than half of the genes displaying proteomics changes also vary significantly within the transcriptome (12/26) (FDR <0.05). Therefore the majority of proteins (14/26) that increased in *puf3*Δ strains did not exhibit a corresponding increase in mRNA abundance, consistent with a putative translational repression role for Puf3p.

The label free mass spectrometry results for two proteins that increase in *puf3*Δ strains for which we had available antibodies, Tim10p and Ssc1p, were confirmed by western blotting. When quantified, antibody signals for Tim10p and Ssc1p increase in protein concentration in *puf3*Δ strains 1.5 fold by western blotting (Fig. 4C) and 1.9/1.7 fold respectively by quantitative label-free MS (Fig. 4A). Our analysis suggests that altered mRNA-abundance in *puf3*∆ can explain only part of the changed protein levels we observe.

### Altered ribosome occupancy of mRNAs in the *puf3*Δ strain

**Figure 5:**
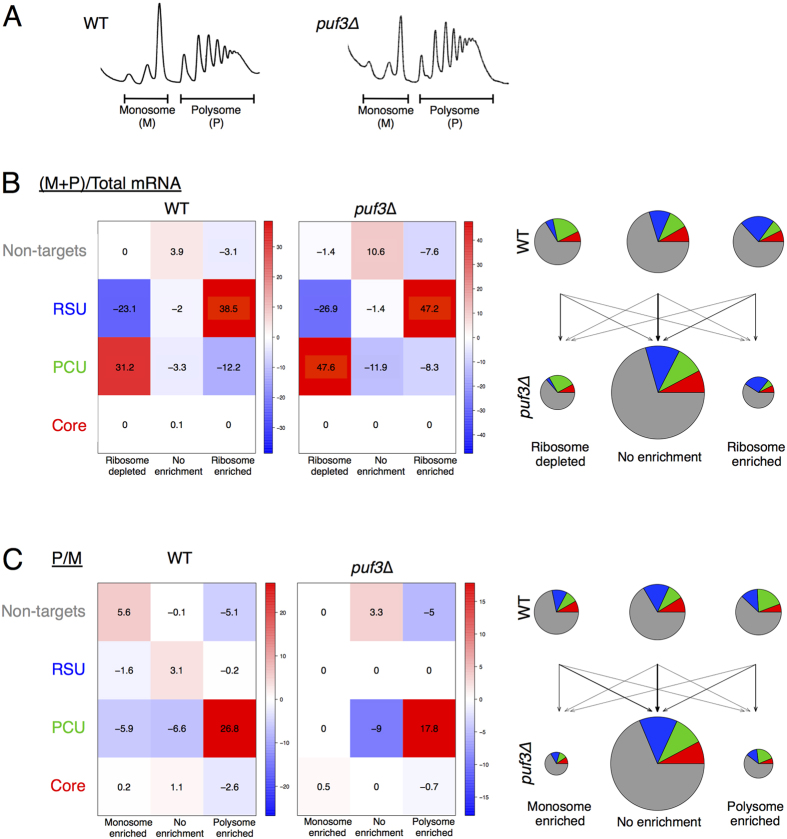
Translatomics comparison of *puf3∆* and wild-type strains. (**A**) Polysome profiles of extracts from wild-type and *puf3*∆ cells, performed in triplicate and resolved on 15–50% sucrose gradients. Regions pooled for monosome (M) and polysome (P) RNA-seq are shown. (**B**) Analysis of the impact of *puf3*∆ on the engagement of mRNAs in the four indicated groups with ribosomes as determined by determining from RNA sequencing the fraction of each mRNA in monosomes (M) + polysomes (P) versus total mRNA for wild-type (WT) (left) and *puf3∆* cells (middle). Each mRNA is designated as enriched, depleted or neither according to the EdgeR analysis (see Supplementary Dataset S1). Statistical enrichments (positive numbers shaded red) or depletions (negative numbers, shaded blue) for each grouping are shown for enrichments where 2 or −2 indicates *P* = 0.01 and 3 or −3 indicates *P* = 0.001 etc. The right panels depict the same data as a series of pie charts showing the relative changes in each grouping in the *puf3*∆ strain. (**C**) Analysis as per panel B except comparing polysome (P) to monosome (M) ratios in each strain. The full datasets are shown in Supplementary Dataset S1.

To further rationalise the observed protein changes, we wished to assess the contribution of translational controls to Puf3p functions in more detail. Puf3p has been shown to co-sediment with polyribosomes^17^ and associate with translation factors^21^, which is consistent with a role in translation and/or RNA decay. We used sucrose gradient separation of mRNAs and pooling of ribosome associated RNAs into monosomal and polysomal fractions (Fig. 5A), prior to RNA sequencing. Variations of this approach have been widely used before^29^,^30^,^31^. Comparing abundance of mRNA in ‘translatome’ fractions (monosomes and polysomes combined, thereby eliminating the mRNA within the ribosome free fractions at the top of each gradient) with the corresponding total transcriptome (Supplementary Dataset S1), reveals how well engaged each mRNA is with ribosomes. Of 5578 mRNAs quantified, we observed 1768 mRNAs enriched in the ribosome-bound fractions of wild-type cells, while 1635 were depleted (or enriched more within ribosome free fractions). The remaining mRNAs are therefore considered to be neither enriched nor depleted in the translatome relative to the transcriptome. In *puf3∆* cells, many fewer mRNAs are differentially engaged with ribosomes than in wild-type cells (1075 enriched and 1193 depleted from 5646 total mRNAs), suggesting that the ribosome engagement of many mRNAs more closely resembles their overall abundance in *puf3*∆ cells and that one role of Puf3p is to enhance differential translation rates between mRNAs.

Analysing the translational response of Puf3p target mRNAs, we found that Core targets are neither enriched or depleted in the ribosomal fractions (monosomes + polysomes) compared with total mRNA. In contrast, the RSU mRNAs are more actively engaged with ribosomes and a significant fraction of the PCU targets are ribosome free (Fig. 5B). These trends are seen in both wild-type and *puf3*∆ cells. Scatterplots of mRNAs highlighting the translational enrichment of the target mRNA subsets are shown in Supplementary Figure S4. The effect of Puf3p appears complex; the ribosome engagement of many mRNAs in *puf3*∆ is increased or decreased, though the mRNAs affected include both Puf3-bound and unbound mRNAs suggesting a ‘second-order’ effect where altered ribosome engagement of targets also affects the ribosome engagement of non-target mRNAs.

Next we compared relative mRNA abundance in the polysome to monosome fractions, an analysis strategy that only considers mRNAs engaged with ribosomes to assess how active translation is in these cells. Polysome/monosome ratios are a frequently used measure of translational activity^30^,^32^. *puf3*∆ has some impact on both Puf3p target mRNAs and non-target mRNAs (Fig. 5C). For example Core targets are depleted from the polysome fraction when Puf3p is present but not in *puf3*∆. The two approaches taken here to analyse the ribosome interactions of each mRNA show that, although complex, both target and non-target mRNA groups each have alterations in ribosome engagement in *puf3*∆ cells.

### Multi-omics hierarchical clustering reveals Puf3 has a modest impact on global gene regulation

**Figure 6:**
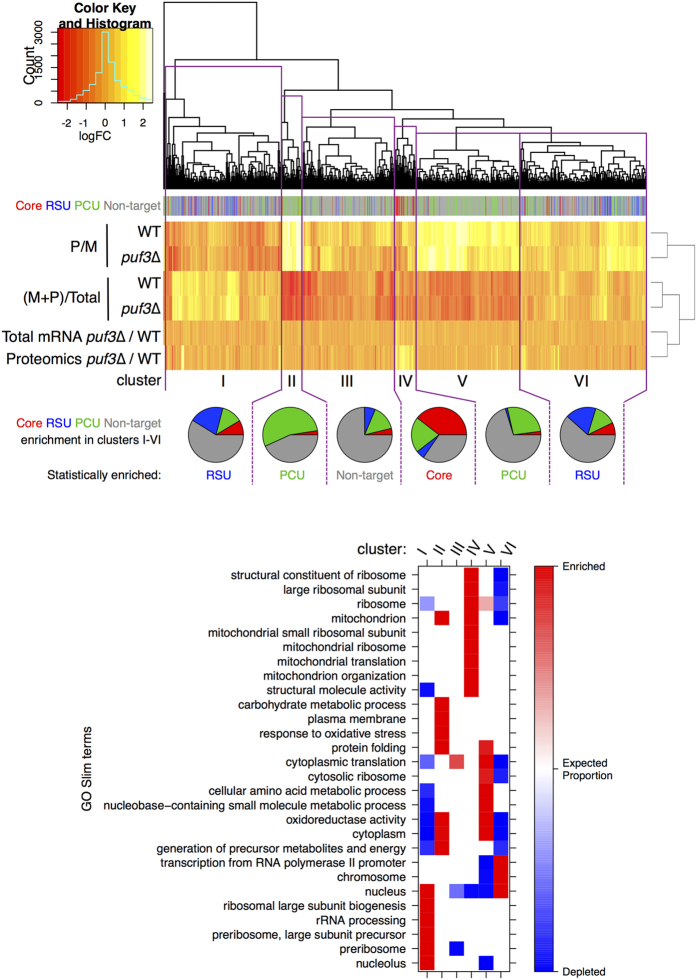
Multi-omics comparison of the impact of *puf3∆* on gene regulation. Hierarchical clustering analyses of log_2_ fold changes from each of our ‘omics datasets analysed in Figures 3, 4, 5. 1798 genes are represented across the 6 data sets. Genes are depicted as coloured vertical lines representing membership of the four Puf3p interacting groups (Core, RSU, PCU and non-target mRNAs). Purple boxes delineate 6 gene clusters (I-VI) with similar profiles. Pie charts show proportional group membership in each cluster. Groups statistically over-represented (*P* < 0.001, Chi squared test) in each cluster are named beneath each pie. Lower panel, GO categories showing a group association (χ^2^ test for independence; Bonferroni corrected *p*-values < .01). Colours show if the GO terms are significantly enriched (red) or depleted (blue) within the groups. White colour indicates non-significant enrichments/depletions. See Supplementary Dataset S1 for a list of genes in each cluster.

Finally, we considered the wild-type and *puf3*∆ transcriptome, translatome and proteome responses of yeast genes in a concerted multi-omic analysis, shown in Fig. 6 as a clustered heat map. Our RIP-seq fold enrichment data was excluded from the clustering as it would impose significant bias, invalidating the results. Instead colour-coding shows gene membership of the independently derived four Puf3p interaction classes as a separate row between the dendrogram and heat map. The proteomics data restricted the number of genes surveyed to ~1800. It was possible to identify 6 broad clusters (bounded in purple boxes labelled I-VI) with consistent expression properties. Cluster IV genes show increased protein levels in *puf3*∆ cells and are significantly enriched in Core mRNA targets of Puf3p (*p* < 0.001). Occupancy of the other cluster-groups reflects coherent patterns of enrichment in the metrics reflecting different aspects of translation, likely dominated by responses to changes in ribosome engagement (rows 1–4 of the heat map). The RSU group are significantly statistically enriched in clusters I and VI, while the PCU genes are enriched in clusters II and V. The clusters also show enrichments in GO terms (Fig. 6, lower panel) following the pattern observed in the corresponding IP groups (Fig. 2). Clusters I and VI are enriched for nuclear functions, Clusters II and V for carbohydrate and amino acid metabolism, while cluster IV, enriched in Core targets, identifies mitochondrial functions. When viewed as a whole, the data reveal that different groups of Puf3p targets partition into sets with different translational properties; however, at steady-state there do not appear to be large-scale differences between the wild-type and *puf3*∆ strains. Therefore for the majority of Puf3p-bound mRNAs loss of *PUF3* has only limited apparent impact on gene expression.

## Discussion

Puf3p binds sequence motifs frequently found within the 3′ UTRs of its target mRNAs. However, mechanistic details of how Puf3p influences the fate of its mRNA targets remain unclear. Much is known about selected genes, such as *COX17*, one of the first identified mRNA targets of Puf3p^7^. It is now well established that Puf3p binding in glucose-grown cells accelerates degradation of *COX17* mRNA^20^,^22^,^33^,^34^. However, is *COX17* a typical target of Puf3p? A recent study used northern blotting to measure mRNA half-lives of ten additional Puf3p target mRNAs^20^. The mRNAs studied were also stabilized when *PUF3* was deleted, however not by the same extent as *COX17*. In the present study we used a series of unbiased genome-wide approaches to define a comprehensive set of Puf3p mRNA targets and determine the impact of *puf3∆* at steady state on the transcriptome and proteome. We have increased the number of Puf3p mRNA targets significantly beyond those identified in prior studies. By comparing our Puf3p targets with previous studies, and analysing their fate in the transcriptome, translatome and proteome of *puf3*Δ strains, we provide a multi-omics view of the role of Puf3p.

The 1132 Puf3p target mRNAs described here correlate excellently with the original RIP-chip study^8^,^9^: 96% of its 225 targets were identified here (Fig. 1A,B). We ascribe our expanded set of targets to the increased dynamic range and statistical power of sequencing compared to microarrays. In support of this, we note that the majority of RSU Puf3p targets were enriched in the Hogan *et al*. dataset^9^ (Fig. 1A), but at lower or non-significant levels. The overlap between our RIP-seq dataset and the PAR-clip study^23^ is lower, at 33%, though it is clear many of the larger RIP-seq fold changes are in common (Fig. 1A,B). Methodological differences between the studies suggest a possible explanation. For example, PAR-clip sample preparation entailed resuspending cells in buffer for an extended incubation during the UV cross-linking procedure^23^. Removing glucose or amino acids causes rapid inhibition of protein synthesis initiation^31^,^35^. Also, as our manuscript was being finalised it was reported that glucose withdrawal alters Puf3p phosphorylation and affects its function^36^. GO term analysis of PCU targets is consistent with this view (Fig. 2), showing an enrichment of carbohydrate metabolic processes which are known early responses to both amino acid and glucose starvation^31^,^32^. We therefore speculate that PCU targets could reflect altered Puf3p binding following glucose starvation, mediated by the recently reported starvation-induced Puf3p phosphorylation^36^.

Like other studies, we observed Puf3p binding to nuclear encoded mRNAs specifying mitochondrial proteins^9^,^23^. Additionally, however, our GO analyses (Figs 2 and 6) imply that Puf3p also targets mRNAs encoding proteins destined for the nucleus. Interestingly, prior studies have reported that Puf3p interacts with nuclear proteins^25^, suggesting Puf3 might shuttle mRNAs to the nucleus, in a fashion similar to its mitochondrial role^18^. This suggests that, dependent on environmental conditions, Puf3p may act to shuttle mRNAs to a variety of organelles, not just mitochondria, an area that could be explored in future studies. One cautionary note to add here is that reported Puf3p protein-interacting partners might be RNA-mediated^21^. Similarly, Puf3p could be interacting with nascent protein chains emerging from translating ribosomes, which while often predictive of protein-protein interactions^37^ may not be functionally important.

A major role assigned to Puf3p is to promote transcript instability, primarily as a result of a series of compelling studies on its impact on *COX17* mRNA^20^,^22^,^33^,^34^. However our RNA-seq experiment (Fig. 3) suggests this is an over-simplification as *puf3∆* apparently affects the abundance of only a limited subset of the mRNAs it binds. Relatively few of the Core mRNA targets increase in steady state abundance, while very few RSU and PCU mRNA levels alter. Indeed, in this context *COX17* appears as an extreme outlier, exhibiting the greatest change in abundance of all mRNAs in *puf3*Δ strains (Fig. 3B). Although recently reported half-life measurements of a selection of Puf3p target mRNAs^20^ correlate well with our steady state abundance data (Fig. 3), this sample of mRNAs does not appear fully representative of the role Puf3p plays on its wider target mRNA population. One clear possibility is that Puf3p plays a dominant role in regulating mRNA stability on some of its targets, such as *COX17*, but acts with other RBPs including translation factors, members of the Lsm complex, the ribosome^21^ and other mRNA specific RBPs, such as Slf1p^38^, to influence RNA biology on a wider set of mRNAs.

As noted above, it was recently suggested that Puf3p may act as a translational activator on some mRNA targets following glucose starvation^36^. Although we did not use starvation conditions, we found previously that Puf3p associates with translating polysomes in glucose-grown cells and it is therefore positioned to regulate translation under our growth conditions^17^. Our proteomics in *puf3*∆ did not detect many significant protein level changes, but the analysis was biased towards abundant proteins. Of those proteins whose levels did change in the *puf3*∆ strain, many from the Core set of Puf3p targets did not exhibit matched changes in steady-state mRNA levels (Fig. 4). These observations are fully consistent with a translational repression role for Puf3p on some of its targets. Our translatomics analyses (Fig. 5) provided additional support for a role of Puf3p in translational control, but indicate that the overall picture is complex. For example, for the 21 Puf3p target proteins quantified by proteomics as increasing in *puf3*∆ (Fig. 4), ten of their mRNAs increased polysome/monosome ratios in *puf3∆* cells compared to WT, including *HSP60*, *MRPL35, SSC1* and *HEM2*. Other targets including *COX17* migrate in the opposite direction, suggesting the increased *COX17* mRNA in *puf3*∆ is not well engaged with polysomes, but still boosts Cox17p levels. However as *COX17* is a short transcript, our polysome profiling analysis lacks the resolution power of the ribosome footprinting technique^39^ to detect modest changes in ribosome occupancy. Overall our data indicate Puf3p has roles both promoting and repressing ribosome engagement of different mRNAs, indicating that Puf3p likely enhances differential translation rates between mRNAs. Thus overall, a complex picture of multi-faceted control via Puf3p is emerging (Fig. 6) that will require further study.

In summary, by employing a series of genome wide technologies we quantitatively assess the role of Puf3p in actively growing cells. RIP-seq of Puf3-TAP suggests that it can bind mRNAs that encode proteins targeted to the nucleus in addition to its well characterised role in mitochondrial biogenesis. Our transcriptomics, translatomics and proteomics suggest that Puf3p controls the abundance of a relatively small proportion of its target mRNAs (eg *COX17*) and points to additional roles for Puf3p, including translational control. Our data provide a resource for future studies and we suggest that applying similar methods to stressed cells may be informative when examining the changing landscape of Puf3p interactions and its effects on gene expression.

## Methods

### Strains and growth conditions

The *PUF3*-TAP-tagged His^+^ strain in the BY4741 background and *PUF5*-TAP were obtained from Open Biosystems. *CDC33*-TAP and an untagged *HIS3* BY4741 strain (GP6001) used as a control for TAP experiments were recently described^40^. BY4741 and its *puf3*Δ::KanMX derivative were obtained from Euroscarf. Cell Growth was performed at 30 °C in either SCD-His, for the TAP strains, or SCD, for the BY4741 and its *puf3*Δ derivative^41^. Cells were grown to A_600_ = 0.6.

### Immunoprecipitation of TAP tagged proteins

Yeast grown in triplicate were lysed into Buffer A (20 mM Tris-HCl pH8, 140 mM NaCl, 1 mM MgCl_2_, 0.5% NP40, 0.5 mM DTT, 1 mM PMSF, EDTA free Protease Inhibitor cocktail tablet (Roche), 100 μM NaV_3_O_4_, 5 mM NaF and 40 units/ml RNAsin) using a 6870 Freezer mill (Spex). Cell lysates were cleared by centrifuging twice at 15,000 g. Beads were pre-washed thrice with Buffer A and then added to 4 mg/ml of lysate. Immunoprecipitations were performed for 20 minutes at 4 ^o^C and washed five times with Buffer A containing 10 units/ml RNAsin, changing tubes twice during the washes and the final two washes were performed for 15 minutes each. For RNA isolation after the final wash the beads were resuspended in 250 μl Buffer A and treated with Trizol. The aqueous phase was mixed with 70% ethanol and the RNA was purified using RNeasy Minikit (Qiagen).

### Transcriptome analysis

The parental and *puf3*∆ strains were grown in triplicate in SCD medium to A_600_ = 0.5–0.6 and treated as described above. RNA was isolated from cleared lysates using Trizol and used to generate sequencing libraries.

### Generation of sequencing libraries

Once isolated, all RNA samples were processed as described previously^40^,^38^. Briefly, rRNA was depleted from the RNA. Sequencing libraries were generated using the whole Transcriptome Library preparation protocol provided with the SOLiD® Total RNA-Seq Kit. Samples were sequenced either on an ABI SOLiD® 4 or an ABI SOLiD® 5500xl.

### RT-PCR

Quantitative RT-PCR was performed in triplicate on samples collected from total and immunoprecipitated RNA samples and amplified using specific oligonucleotide primers designed to the indicated transcripts primers as described previously^17^.

### Ribosome co-sedimentation analysis

Polyribosomal profiling was performed as previously described^42^. Briefly, *S. cerevisiae* was grown in triplicate to an OD_600_ 0.7, cycloheximide was added to a final concentration of 0.1 mg/ml and yeast were harvested by centrifugation. *S. cerevisiae* were lysed into polyribosomal buffer containing cyclohexamide and either 2.5 OD_260_ units or 9 OD_260_ units were loaded onto a sucrose gradient. 15–50% sucrose gradients were poured as previously described^42^. 15 fractions were collected from each gradient. For the genome-wide analysis of translation factions 4–8 were pooled to form the monosome samples and 10–15 were pooled to form the polysome samples.

### Computational biology methods

Processing of SOLiD Sequencing data, RNA-Protein Network Analyses and Motif discovery are described in the Supplementary Methods.

### Label-free protein quantification

Quintuplicate repeats of the wild-type and *puf3*Δ strains were grown in SCD medium to exponential phase. Cultures were harvested and processed and analysed by LC-MS/MS using an UltiMate^®^ 3000 Rapid Separation LC (RSLC, Dionex Corporation, Sunnyvale, CA) coupled to an Orbitrap Elite (Thermo Fisher Scientific, Waltham, MA) mass spectrometer as previously described^38^,^43^. The acquired MS data from the five replicates were analysed using Progenesis LC-MS (v4.1, Nonlinear Dynamics).

### Western Blotting

SDS-PAGE and immunoblotting used standard techniques with Enhanced chemiluminescent detection. Antibody signals to Ssc1p (1:5000), Tim10p (1:1000) and Tef1p (1:2000) were quantified using National Institutes of Health Image J software. Ssc1 and Tim10 antisera were a kind gift from Kostas Tokatlidis.

## Additional Information

**Accession codes**: Sequencing data are publicly available at ArrayExpress; E-MTAB-3406, EMTAB-3407, and E-MTAB-3413. The mass spectrometry proteomics data have been deposited to the ProteomeXchange Consortium (http://www.proteomexchange.org) via the PRIDE partner repository with the dataset identifier PXD001903 and DOI 10.6019/PXD001903.

**How to cite this article**: Kershaw, C. J. *et al*. Integrated multi-omics analyses reveal the pleiotropic nature of the control of gene expression by Puf3p. *Sci. Rep*. **5**, 15518; doi: 10.1038/srep15518 (2015).

## Supplementary Material

Supplementary Information

Supplementary Dataset S1
