# Supplementary material for: Integrated multi-omics analyses reveal the pleiotropic nature of the control of gene expression by Puf3p
Source: Sci Rep. 2015 Oct 23;5:15518. doi: 10.1038/srep15518 (PMC4616039; doi:10.1038/srep15518)

SUPPLEMENTARY MATERIAL

Integrated multi-omics analyses reveal the pleiotropic nature of the control of gene expression by Puf3p

Christopher J. Kershaw[1], Joseph L. Costello[1,2], David Talavera[1], William Rowe, Lydia M. Castelli, Paul F. G. Sims, Christopher M. Grant, Mark P. Ashe, Simon J. Hubbard* and Graham D. Pavitt*.

Index

Supplementary Dataset S1	page 1
Supplementary Results Text S1	page 2
Supplementary Methods	page 5
Supplementary Additional References	page 6
Supplementary Figure Legends	page 7
Supplementary Figures S1-S4	page 9-12

Supplementary Dataset S1

Datasets in MS excel format. A series of 13 'Tables' is presented in a separate single Microsoft excel file providing processed RIP-seq, RNA-seq and proteomics data and analyses. See the dataset header sheet for details.

Supplementary Results Text S1

To investigate whether the 720 RSU targets represented *bona fide* Puf3 candidates or were enriched in our data for other reasons. Three possible sources of indirect positive interactions were considered:

1. To address non-specific binding by the IgG-coupled beads, we performed additional control TAP-IPs using an untagged strain, Puf3-TAP, Puf5-TAP and eIF4E-TAP and amplified specific mRNAs using an endpoint RT-PCR approach. Puf5p is a related PUF protein that binds to distinct mRNAs through a motif related to the Puf3-binding motif[8,9], while eIF4E is a general mRNA 5'cap-binding protein that is important for translation initiation[40]. PCR of the Puf3-TAP IP amplified both the prototypical Core Puf3 target RNA *COX17* and *CBP3*, a novel target only identified by our RIP-seq study, but did not amplify *PGK1* an example mRNA identified by PAR-clip or other control mRNAs. Similarly Puf5-TAP bound only its target *ORC2,* while the untagged strain failed to amplify any products, while all mRNAs tested were found to bind to eIF4E-TAP, as expected (Supplemental Figure 2A). This analysis confirms that our experimental approach can isolate specific mRNAs.

   In addition, we were unable to purify sufficient RNA from an untagged strain to perform sequencing. As a further test for non-specific binding to our affinity matrix, we thought that the same mRNAs would likely bind non-specifically and would be enriched in other IPs performed using the same technology. We have recently performed equivalent RIP-seq experiments on eight translation factors and RNA-binding proteins[38,40]. By comparing to these previous RIP-seq experiments we found that no mRNAs were universally enriched in these previous datasets (FDR < 0.01).

2. Although Puf3p did not bind the Puf5p target *ORC2,* we also considered indirect binding to other PUF protein targets via Puf3p as a possible explanation. We compared the overlap between Puf3 RIP-seq targets and those RNAs identified previously as Puf1p Puf2p, Puf4p or Puf5p targets by RIP-chip[8,9] (Supplemental Figure 2B). Other than Puf3p targets, no significant overlaps were seen, meaning that capture of other Puf-target mRNAs does not explain our RSU set.

3. Finally, to address possible indirect binding by Puf3p to the 720 RSU mRNAs via other co- precipitating factors, we examined whether other protein-protein interactions and other RBP-mRNA interactions reported in the BioGRID database[25] might offer an alternative explanation for the presence of these transcripts in our data set. This was done in order to predict the most direct mechanism by which Puf3p could pull down the RSU targets. We defined all reported interactions where Puf3p directly binds mRNAs as first order interactions (which mostly comprise the PAR-clip dataset, as these data are included in BioGRID). If no direct binding was reported, but there was evidence that Puf3p binds another RBP, which subsequently binds mRNAs, these interactions were classified as second order interactions. Consequently, third or higher order interactions need two or more intermediate proteins, respectively to bridge the interaction. In all cases, each Puf3p-mRNA interaction was classified with the lowest possible order. As expected, the Core and PCU targets are predominantly first order binders (Supplemental Figure 2C), since most are directly reported in BioGRID from previous studies[9,23]. In contrast the novel RSU targets are, at best, potential third order or higher order interactions, similar to non-Puf3p targets. The absence of second order interactions suggests that the RSU targets can not be explained simply as a result of indirect binding via other known protein partners and their associated RNAs.

Additionally, we checked if any protein-protein interactions with other RBPs might cause the misidentification of Puf3p targets. We found that RBPs that bind any of the RSU targets also bind many non-Puf3p targets. By these independent measures we suggest that the 720 mRNAs comprise novel Puf3p targets.

Supplementary Methods

Processing of SOLiD Sequencing data

Reads were mapped to the *S. cerevisiae* genome (genome assembly EF4 downloaded from ENSEMBL) using Bowtie; sequences were then assigned to genomic features using HTseqcount (mapping against the corresponding EF4 GTF file). Sequencing data are publicly available at ArrayExpress; E-MTAB-3406, E-MTAB-3407, and E-MTAB-3413.

Transcript enrichment/depletion analyses were performed using different tests implemented in the edgeR package[44]. Enrichments were tested for using the Fisher test, and applying the Benjamini and Hochberg correction to the calculated *P*-values. The contrasts between the transcriptome and the monosome or polysome fractions were performed using the exact test in the classical approach. In addition, we compared the transcriptome counts to the average of monosome and polysome counts (translatome counts). We used the generalized linear model (GLM) approach for this analysis. We also used the GLM approach when we compared the monosome and polysome fractions, as we had an experimental design with paired samples. Functional enrichment analyses were performed in-house. GO-Slim mapping annotations were downloaded from the Saccharomyces Genome Database (www.yeastgenome.org).

RNA-Protein Network Analyses

Physical and genetic interactions were downloaded from the BioGRID database (version 3.2.111). In order to study if indirect binding could cause the pull down of some mRNAs, we performed graph analyses where we counted the number of Puf3p targets that could be explained by first, second, third or higher order interactions according to current knowledge. Additionally, we analysed the importance of unreported Puf3p-RBP-RNA interactions. For each RBP with known RNA targets, but not known to bind Puf3p, we assumed that an interaction could be identified in the future. Then, we compared the number of Puf3p target

RNAs that could be explained by indirect binding this way with the number of non-targets that would conflict with the indirect binding hypothesis.

Motif discovery

MEME (version 4.10.0) was run locally to identify commons motifs[24]. In order to increase the discriminative power of the tool, we used the set of non-targets as a negative set for calculating position-specific priors. We used UTR sequences reported in RNA-Seq experiments[45]. For Core targets, the motif was found in 201 out of the available 204 3' UTRs. The reported motif E-value was 2.3×10^{-187}. 3' UTR sequences were available for 183 RSU targets and the motif was found in all 183 3' UTRs, which a corresponding motif E-value of 4.0×10^{-11}. During an exploratory phase more than one motif was considered, but no additional motifs returned were significant. We also looked for motifs in 5' UTRs and a selection of ORF sequences, but we did not find any motif with a low E-value and/or present in most of the input sequences.

Supplementary Additional References

44. Robinson, M.D., McCarthy, D.J. & Smyth, G.K. edgeR: a Bioconductor package for differential expression analysis of digital gene expression data. *Bioinformatics* **26**, 139-140 (2010).

45. Kertesz, M. et al. Genome-wide measurement of RNA secondary structure in yeast. *Nature* **467**, 103-7 (2010).

Supplementary Figure S1. Cluster analysis of Puf3-TAP IP and Total RNA sequencing. Diagram shows that the biological replicates cluster together and that the IP samples are distinct from the Total mRNA samples.

Supplementary Figure S2. Novel RIP-seq mRNA targets are not explained by indirect interactions. (**A**) Specificity of TAP IP is demonstrated by agarose gel images of products of RT-PCR reactions with primers designed to selected mRNAs following TAP affinity chromatography of the indicated strains. (**B**) Dendrograms and keys comparing RIP-chip RNA targets for each PUF protein[9] with our Puf3-TAP RIP-seq (q-value < 0.01). Left panel shows all 1132 targets, while the right splits the Puf3p data into Core, RSU, PCU and non-targets. (**C**) Histogram describing the lower order interactions that can explain targets for each datasets as determined by interactions captured within the BioGRID database[25] shows RSU class cannot be explained by known secondary protein-protein and protein RNA interactions of Puf3p. (**D**) Plots of log10 RPKM (reads per kilobase per million) from the PAR-clip study data[23] highlighting the relative abundance of Core (red), RSU (blue) and PCU (green) mRNA targets as defined in Figure 1A.

Supplementary Figure S3. Notched Box and whisker plots comparing attributes of Puf3p mRNA targets. Histograms above each plot show binned total data with the vertical dashed line indicating the median of the total. Box-and-whisker plots depict different datasets. A 95% confidence interval around the median is represented by a notch. Where notches do not overlap the medians differ. The right and left lines of the box are the 3rd and 1st quartiles with the length of the rectangle the interquartile range (IQR). The right whiskers denote Q3 + 1.5xIQR, similarly the left whiskers denote either the minimum value or Q1 – 1.5xIQR, whichever is larger. Outlier points are shown as open circles. The existence of differences was

firstly examined using the Kruskal-Wallis test. Where a significant difference was found, gene sets were statistically tested versus the set of mRNAs not bound in any dataset. *P* values represent FDR (Mann-Whitney U tests corrected for multiple hypothesis testing). Datasets used for comparisons are (**A**) Median poly A tail length[26] (**B**) mRNA half-life[27].

Supplementary Figure S4. Translatomics analysis. Plots of log2 fold change (monosome + polysome)/total RNA for wild-type (x-axis) verses *puf3*Δ strain (y-axis). The first plot highlights Core, RSU and PCU sets together, while the other three plots just a single gene set. Note PCU set (green) are shifted down and left indicating depletion from ribosomes, while RSU (blue) are shifted up and right indicating enrichment with ribosomes.

Supplementary Figure S1

Puf3-TAP, IP v total RNA

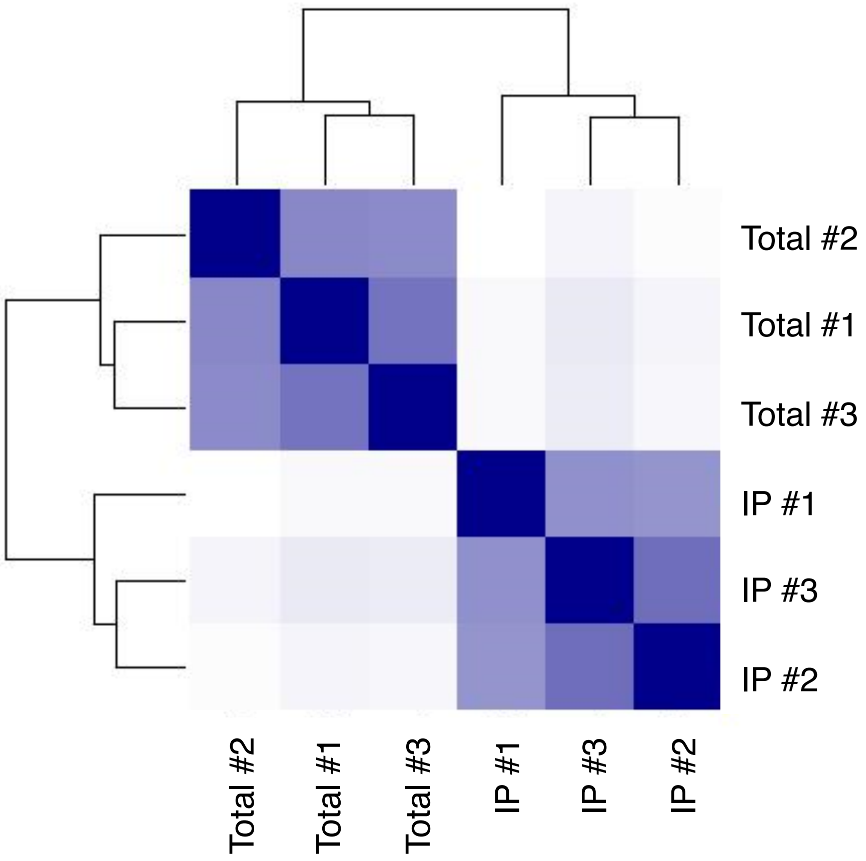

Supplementary Figure S2

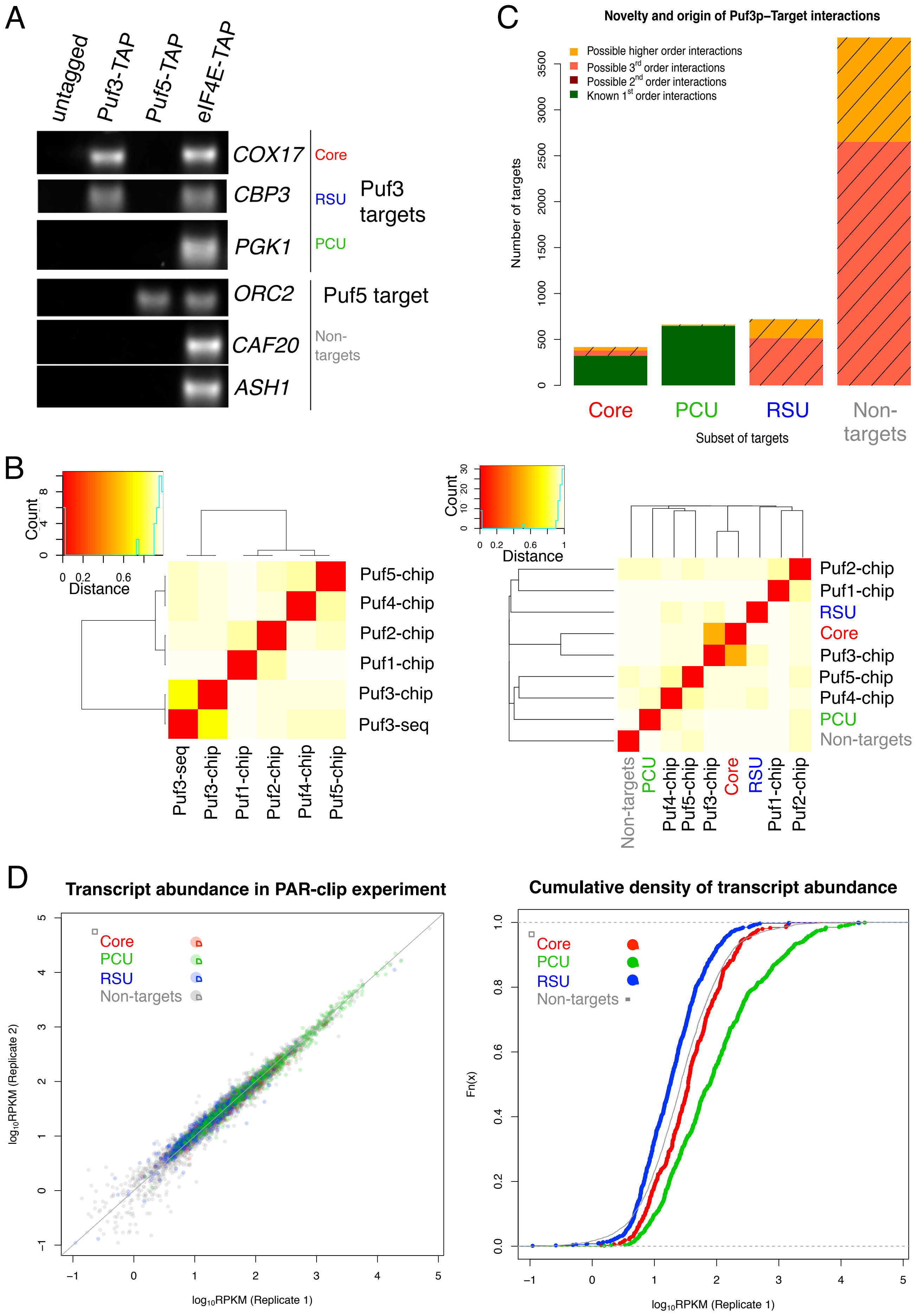

Supplementary Figure S3

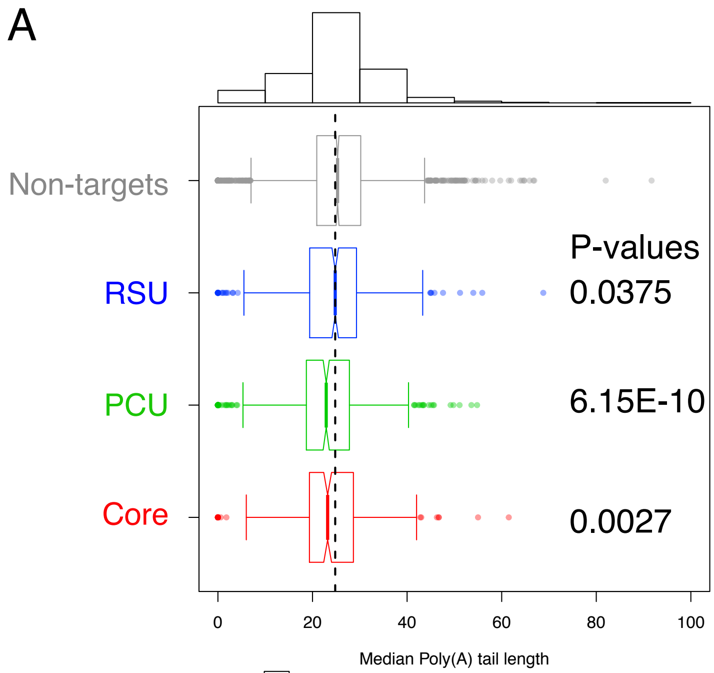

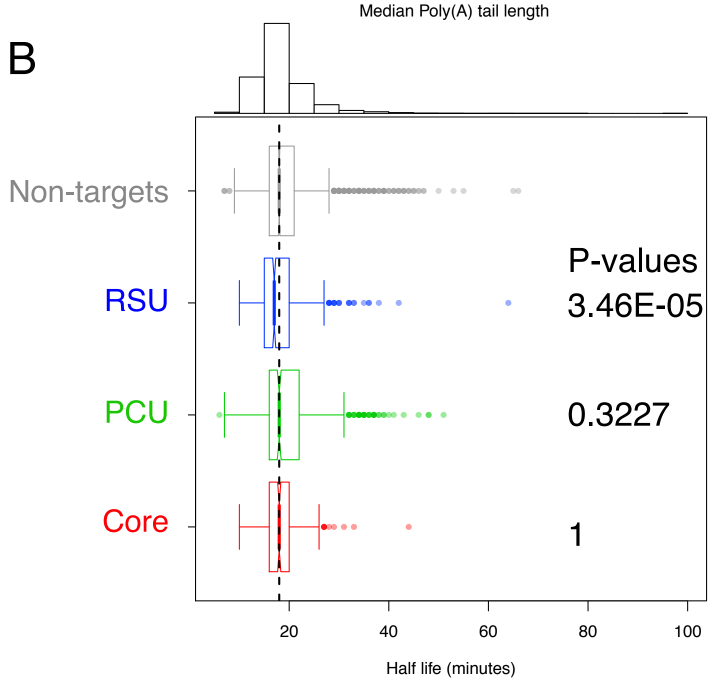

Supplementary Figure S4

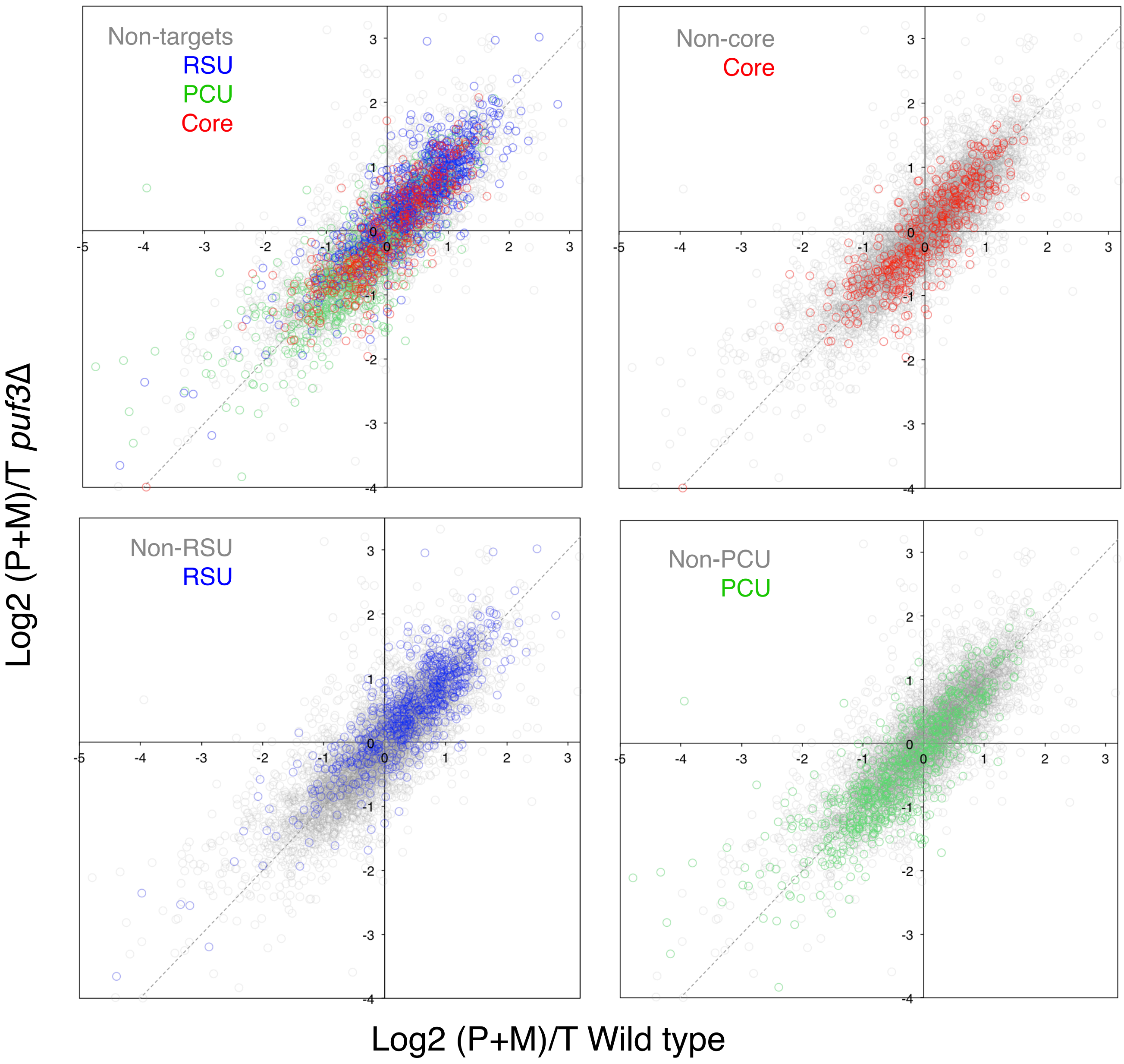

Supplement: Supplementary Information [file srep15518-s1.pdf]
